# Nursing Documentation Practices and Related Factors in Patient Care in Ethiopia: A Systematic Review and Meta-Analysis

**DOI:** 10.1155/2023/5565226

**Published:** 2023-11-06

**Authors:** Temesgen Geta Hardido, Beimnet Desalegn Kedida, Eustes Kigongo

**Affiliations:** ^1^School of Nursing, Wolaita Sodo University, Wolaita, Soddo, Ethiopia; ^2^School of Public Health, Wolaita Sodo University, Wolaita, Soddo, Ethiopia; ^3^Faculty of Public Health, Lira University, Lira, Uganda

## Abstract

**Background:**

Ineffective nursing documentation practices have been reported to negatively impact patient outcomes and health professional efficiency. On the prevalence of nurses' documentation practices in Ethiopia, several separate studies have been carried out. However, there is no pooled prevalence of nurses' documentation practice. Therefore, this systematic review and meta-analysis aimed to assess the overall prevalence of nursing care documentation practice and related factors in Ethiopia.

**Methods and Materials:**

This review only included articles that were published. The main databases were Medline/PubMed, Web of Science, Google Scholar, Scopus, Ethiopian University Repository Online, and the Cochrane Library. Cross-sectional studies that satisfy the criteria and are written in English are included in the review. Using a random effects model, the pooled prevalence of nurses' documentation practices was determined. The funnel plot and the Eggers test were also used to look into publication bias. All statistical analyses were done with STATA version 14.

**Result:**

This review included nine studies with a total of 2,900 participants. The pooled prevalence of nurses' documentation practice in Ethiopia was 50.01% (95% CI: 42.59 and 57.18; *I*^2^ = 93.8%; and *P* ≤ 0.001). In terms of subgroup analysis, Addis Ababa had the highest prevalence of nurses' documentation practice at 84% (95% CI: 77.18 and 90.82), while Southern Ethiopia had the lowest at 40.00% (95% CI: 38.10 and 44.90). Nursing documentation practices were statistically associated with the availability of nursing documentation formats, adequate nurse-to-patient ratio, motivation, and training.

**Conclusion:**

This review showed that one in two nurses practiced poor documentation of their daily activities in Ethiopia. Therefore, strict monitoring, evaluation, and supervision of nursing care documentation services are highly recommended for all stakeholders. We strongly recommend improving the identified factors by arranging training for nurses, motivating them, providing adequate documentation formats, and maintaining a nurse-to-patient ratio.

## 1. Introduction

A detailed record keeping system that is comprehensive, accurate, and timely is required for good nursing practice. Without complete recording, there is no evidence to prove that care was provided to the patient. It is said that “what is not recorded has not been done” [1, 2]. Nursing documentation is a record of care planned and delivered to an individual patient/client that meets legal and professional requirements [3–5]. Documentation of care may not be everyone's favorite aspect of patient care, but the completeness of all patient records is essential to ensure accurate communication between clinical team members and healthcare providers. Nurses should record both the actions they take and the patient's needs and reactions to the illness and the care they receive [3, 6, 7].

Accurate and comprehensive nursing care documentation is a valuable source of data for data coding, health research, evidence, and rationale for funding and resource management [6, 7], and it is frequently used to evaluate nursing professional practice as part of quality assurance mechanisms such as performance reviews, audits, accreditation process, legislative inspections, and critical incident reviews [6, 8]. All documentation should be recorded in a professional capacity by a nurse in relation to the provision of care. This documentation may include written or electronic health records, audio or videotapes, images, observational charts, checklists, and any other type of documentation related to the patient's care [3, 7, 8].

Poorly documented care can result in longer patient hospital stays, poor communication among medical teams, increased patient mortality, increased medical legal risk, impeded clinical care decisions, incorrect treatment decisions, and poor patient care. Inadequate nursing care documentation services can lead to nurses being dislicensed, loss of income, and reimbursement, impacting patient management and continuity of care, medication errors, and unfavorable patient outcomes [7–11].

According to the South African Nursing Council's analytical report from 2003 to 2008, 769 nurses were convicted of occupational misconduct, of which 587 professional nurses recorded nursing activities in patient records have been accused of not doing so [12]. Keeping medical records is part of a nurse's professional obligations, but many studies have examined deficiencies in the documentation habits of nurses around the world [13].

There is evidence that poor communication among healthcare professionals is a cause of medical errors and patient deaths. Evidence from the United States suggests that documentation errors are responsible for 100,000 deaths and 1.3 million injuries annually [11]. Other evidence also shows that medical errors cause about $20 billion in losses each year [6]. The global trend of neglected, inadequate, and incomplete care records is alarming. Most developing countries, such as Ethiopia, suffer from staffing shortages and, at the same time, an increase in overwork, so the trend of misdocumentation cannot be ignored [1]. Ethiopia has developed strategies such as the Federal Ministry of Health's Nursing Care Operating Standards, which state that the care provided must be clearly and accurately documented. This strategy will help improve the quality of records for the delivery of safe, effective, patient-centered, timely, efficient, and equitable healthcare services [14].

Especially in developing countries like Ethiopia, patient medical records are generally poorly supported and poorly managed despite the importance of documentation for quality and efficient nursing care management. However, documentation is not a priority. According to the literature of previous studies, the documentation practice of nurses was 75% in Nepal [15], 74% in Ghana [16], 47.5% in Harar in Ethiopia [17], and 37.4% in Gondar [18]. Poor documentation practices are influenced by a variety of factors, including nurses' attitudes and knowledge, experience, workload, education, motivation, and nurse-patient relationships [14–18]. Accurate and up-to-date documented information is important not only for providing quality care but also for continuing and maintaining medical care at optimal levels. Recent and general information on nurse documentation practices and related factors is potentially of great importance, especially for the development of the health sector in Ethiopia [19, 20].

Although several separate studies have been conducted to determine the level of practice of documentation by nurses in different regions of Ethiopia, there is no national study that demonstrates the overall practice of documentation of nurses on patient care. Also, no single study's representativeness or results are conclusive or consistent. The aim of this systematic review and meta-analysis was, therefore, to assess the consolidated prevalence of nurse records on patient care and related factors in Ethiopia. The results of this study lead to general insights that can help reduce poor nurse documentation practices by supporting the development of guidelines and design strategies. This plays an important role in mitigating adverse patient outcomes.

## 2. Methods and Materials

### 2.1. Search Strategy

Databases including Medline/PubMed, Web of Science, Google Scholar, Scopus, the Online University of Ethiopia Repository, and the Cochrane Library were used to search for studies from 1 to 30 July 2023. In addition, missing data were redacted by contacting the appropriate authors. We checked the database https://www.crd.york.ac.uk/prospero and the Cochrane Library to ensure the review had not been done before and to avoid duplication. PROSPERO also registered this review with the registration number of CRD42023454700. After confirming there was no similar review previously conducted in Ethiopia, a comprehensive search strategy was developed using multiple Boolean operators through standard population comparison and exposure outcome (PEO) questions. The words “OR” and “AND” were used as search terms. The terms “documentation” OR “medical record” OR “nurses” OR “health care professional” AND “associated factors” OR “influencing factors” AND “Ethiopia” are searched using Boolean operators between 2013 and 2023 are included in this review. All articles retrieved from the database are checked for titles and abstracts before being exported to the EndNote library. These articles met the criteria for inclusion in terms of titles and abstracts that were read in full. Three authors (TG, EK, and BD) performed the search strategy. We strictly follow the PRISMA (Preferred Reporting Items for Systematic Review and Meta-Analysis) protocol to conduct this review.

### 2.2. Eligibility Criteria

#### 2.2.1. Inclusion and Exclusion Criteria

This study included studies conducted in a cross-sectional study designed and published in English. It also included studies conducted from 2013 to 2023. Studies that did not address the prevalence of nurse documentation were excluded. This review excluded studies conducted outside of Ethiopia and study designs other than cross-sectional studies.

### 2.3. Data Extraction

PRISMA was used to select articles for this review. Parameters used to extract data included author's name, publication year, study area, sample size, study population, study design, and results. A Microsoft Excel spreadsheet was used to collect the required data from the accepted papers. Three authors (TG, EK, and BD) independently extracted information from the supplementary documents. Studies meeting the approval requirements were included and tabulated after detailed discussion and discussion of data extraction.

### 2.4. Assessment of Risk of Bias and Quality Assessment

Significant analyzes were performed using the Joanna Brings Institute Review meta-analysis and statistical evaluation tools to assess study quality. Joana identifies research and paper abstracts to decide whether they should be included. Article quality was assessed before selection for final review. Cross-sectional studies were assessed considering the adequacy of the source population, sample size, data collection methods, data collection tools, statistical analyses, and response rates and were scored on a scale of 1–9 points. A study is considered a low risk if it has a quality metric score of 7 or higher (Table 1).

### 2.5. Data Processing and Analysis

A Microsoft Excel spreadsheet was used to extract the data and STATA version 14 was used to analyze the data. A random effects model analysis was used to calculate the pooled prevalence of nurse documentation in Ethiopia. Publication bias was tested using funnel charts and visual analysis. Study heterogeneity was tested using the Cochrane Q-Static and I^2^. Nursing document prevalence in each region was compared with the estimated prevalence using subgroup analyses. A forest pilot format with 95% CI was used to present the pooled prevalence.

## 3. Result

### 3.1. Identification and Characteristics of Included Studies

**Figure 1:**
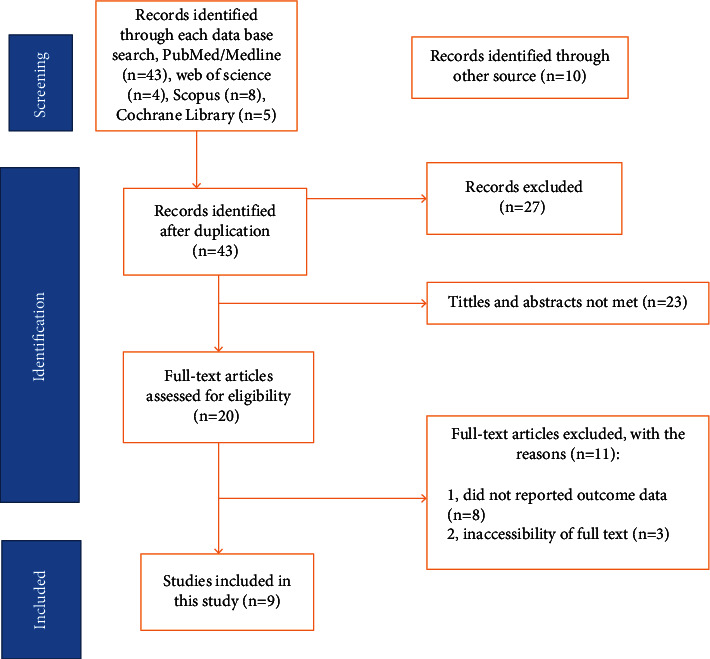
PRISMA flow diagram of study selection for systematic review of nurses' documentation practice in Ethiopia, 2023 (*n* = 9).

From 1 July to 30 July 2023, 70 articles were identified in major electronic databases and other applicable sources. Of these identified items, 27 items were eliminated due to duplication and 43 items were retained for further consideration. Twenty three studies were excluded because the abstract and title did not meet the requirements. Of the remaining 20 papers, 11 studies were excluded due to inconsistencies with the inclusion criteria established for that study. Finally, nine studies that met the eligibility criteria were included in this study (Figure 1).

A total of 9 articles with 2,900 participants were included in this systematic review and meta-analysis. All included studies were cross-sectional studies and the sample size was between 111 [21] and 430 [22]. Regarding the regional distribution of the included studies, two were located in the Oromia region [14, 23], three in the Amhara region [18, 24, 25], and one in the Southern Nations, Nationalities and Peoples Region (SNNPR) [26], Somalia [17], Addis Ababa [21], and Tigray [22] (Table 2).

### 3.2. Practice of Nurses' Documentation of Patient Care in Ethiopia

**Figure 2:**
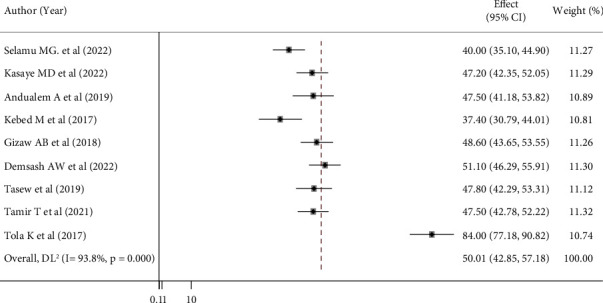
Forest plot showing the pooled prevalence of nurses' documentation of patient care in Ethiopia (*n* = 9).

According to this review, patient care documentation practices by nurses ranged from 37.4% [18] to 84.0% [27]. The pooled estimated prevalence of nurse documentation for patient care in Ethiopia was 50.01% (95% CI: 42.59 and 57.18; *I*^2^ = 93.8%; and *P* ≤ 0.001) (Figure 2).

### 3.3. Subgroup Analysis of Nurses' Documentation Practice in Ethiopia

**Figure 3:**
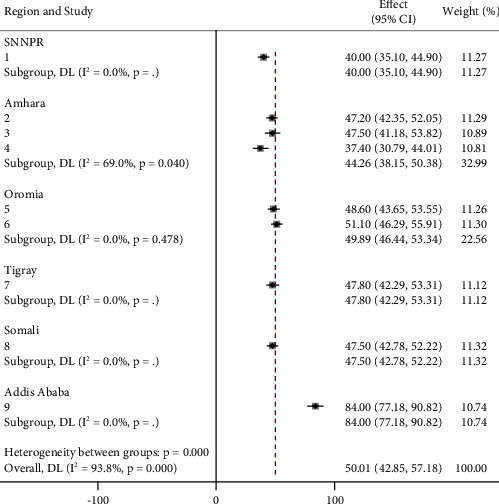
Subgroup analysis of nurses' documentation practice of patient care by region in Ethiopia (*n* = 9).

Subgroup analyzes performed for each region showed that Addis Ababa had the highest prevalence of nursing records at 84.00% (95% CI: 77.18 and 90.82) and Oromia at 49.89% (95% CI: 46.44 and 53.34) followed. The lowest value was observed in the SNNPR region at 40.00% (95% CI: 38.10 and 44.90) (Figure 3).

### 3.4. Heterogeneity and Publication Bias

**Figure 4:**
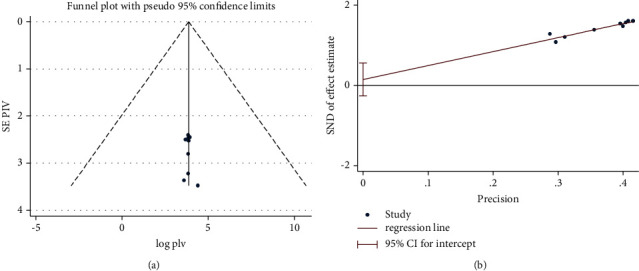
(a) Funnel plot and (b) Eggers test of the review.

To minimize and balance study heterogeneity, subgroup analyzes were performed by region. The results of the *I*^2^ test show that there was significant heterogeneity between studies (*I*^2^ = 98.7%, *P* value  ≤  0.001). Publication bias of the studies was monitored using the Eggers test and visual inspection of funnel charts. Funnel chart results showed that the included studies had a symmetrical distribution after inspection and Egger's test (*P* value = 0.46). This indicates no potential bias (Figures 4(a) and 4(b)) (Table 3).

### 3.5. Outcome

The first interesting result of this systematic review and meta-analysis was the estimate of the integrated prevalence of nurse documentation in Ethiopia. In addition, a combined calculation of the prevalence of nursing documentation in Ethiopia found that one of two Ethiopian nurses practiced poor patient care documentation. A second interesting finding is a factor related to nurse documentation. Results indicated that format availability, appropriate caregiver-patient ratio, motivation, and training likely contributed to good documentation practice.

### 3.6. Factors Associated with Nurses' Documentation Practice in Ethiopia

According to this review, four variables (nurse-to-patient ratio, motivation, practice, and availability of formats) were significantly associated with nurses' documentation practice, while nurses' knowledge (*P*=0.906) and attitudes toward documentation (*P*=0.081) were not significantly related to the level of documentation practice.

The adequate nurse-to-patient ratio was statistically significant in nurse documentation practices (OR = 4.18, CI: 2.5 and 20.59, *P* ≤ 0.001, and *I*^2^ = 88.3%). Nurses who used available documentation forms were three times more likely to provide adequate documentation than nurses who did not use documentation forms (OR = 3.01, CI: 2.03 and 23.07, *P* ≤ 0.001, and *I*^2^ = 93.9%). This review found a significant association between nurse motivation and documentation practices (OR = 26.96, CI: 12.8 and 47.7, *P* ≤ 0.001, and *I*^2^ = 90.9%). Highly motivated nurses were 27 times more likely to practice good documentation compared to their contrast group nurses. Trained nurses were 10 times more likely to practice documentation well than untrained nurses (OR = 10.08, CI: 2.44 and 41.7, *P* ≤ 0.001, and *I*^2^ = 80.9) (Table 3).

## 4. Discussion

Good practice of nursing documentation is essential for routine follow-up and treatment of patients/clients [3, 6]. This systematic review and meta-analysis found that 50% of nursing services in Ethiopia are well documented by nurses (*I*^2^ = 98.7% and *P* < 0.001). This result was higher than studies in Nepal (75%) [15], Ghana (74%) [16], South Africa (68.3%) [28], Nigeria (70%) [1], Jamika (98%) [27], and Iran (100%) [29]. This difference is due to differences in geographic regions, differences in national educational development, policies and strategies, nurses' knowledge and attitudes towards nursing record practice, the availability of administrative support, organizational structure, and methodologies. Compared to this review, findings from Indonesia (37%) [30] and Europe (28%) [31] reported lower results. The possible reason for this discrepancy might be related to variation in methodological differences, particularly the study design and study period.

The review also reported on nurses' documentation practices across the region. It also documented nurses' documentation practices in the region. A subgroup analysis revealed that 84% of the nurses documented their practices in Addis Ababa while a low percentage (40%) of nurses documented their practices in SNNPR. Possibly, the reasons include issues about quality of nursing care, experiences and education among nurses, accessibility and availabilities of formats, high supervision especially from the Ministries of Health being located in Addis Ababa, and others pertaining to the management at the state level. This helps in improving of quality nursing care documentation and supplies.

This systematic and meta-analysis identified a number of factors associated with good nursing documentation practice, including adequate nurse-to-patient ratios, format availability, training, and motivation in nursing documentation practice. The nurse-to-patient ratio was statistically significant for nurse documentation practice. Adequate nurse-patient ratios were 4.18 times more likely to practice good nursing documentation practice than the opposite group. This finding is consistent with studies conducted in Jamaica [27] and Ethiopia [17, 25]. A possible reason for this is that nurses tend to use standard nursing documentation guidelines more often when they have sufficient time for documentation and when patient care is not overcrowded. In addition, one study showed that nurses with low patient burden experienced less stress and busy situations were less likely to be interrupted by other patients when recording activities [32].

Nursing care documentation training was statistically associated with nursing professional's documentation practices. Trained nurses were ten times more likely to document frequent daily care activities than untrained nurses. This review is supported by previous studies [30, 33]. Training may improve nurses' knowledge, attitudes, and teamwork in nursing documentation tasks. They may also become accustomed to standard documentation tools and record their work frequently. This means that all nurses should receive nursing documentation training and improve their documentation practices [17, 34, 35].

Nurse motivation was identified as a factor associated with good practice in nursing records. Highly motivated nurses were more likely than less motivated nurses to have good nursing record practices. This report was consistent with studies conducted in Ethiopia [14, 17, 23]. Evidence suggests that motivated employees develop a positive attitude and documentation practices are carried out responsibly. In contrast, unmotivated caregivers may refuse to continue with their daily activities and affect healthcare outcome [24, 36]. Therefore, all nurses must be motivated to maintain good records of their frequent duties [24].

The availability of standard formats for nursing documentation was positively correlated with the level of nursing staff documentation practice. Previous studies conducted in Jamaica [27] and Australia [37] reported similar results to this finding. Effective nursing documentation practice is supported by the availability of nursing documentation formats [24]. The standardized documentation of patient care requires evidence-based standard formats for data recording [38]. In addition, an easily accessible and available nursing documentation format encourages good documentation practices. This is intended to improve nursing record practice. Appropriate treatment documentation should be available in healthcare facilities.

## 5. Conclusion

This systematic review and meta-analysis revealed that the pooled estimated prevalence of nurse documentation in Ethiopia was 50%. Rigorous monitoring, evaluation, and oversight of national care documentation services are strongly recommended for all concerned bodies. The availability of nursing documentation formats, adequate nurse-patient ratios, motivation, and training were also found to be statistically associated with nurse documentation practices. Therefore, all responsible bodies, such as the Ethiopian Ministry of Health and other stakeholders, should provide training to nurses, motivate nurses, supply adequate documentation formats, and maintain the nurse-patient relationship.

## Figures and Tables

**Table 1:** Critical appraisal results of eligible studies in this study on nurses' documentation practice in Ethiopia, 2023 (*n* = 9).

Selamu et al.	Q1	Q2	Q3	Q4	Q5	Q6	Q7	Q8	Q9	Total
Kasaye et al.	Y	Y	Y	Y	Y	Y	Y	Y	Y	9
Andualem et al.	Y	Y	Y	N	Y	Y	Y	Y	Y	8
Kebed et al.	Y	Y	N	Y	Y	Y	Y	Y	Y	8
Gizaw et al.	Y	Y	Y	Y	Y	Y	Y	Y	Y	9
Demsash et al.	Y	N	Y	Y	Y	Y	Y	Y	Y	8
Tasew et al.	Y	Y	Y	Y	Y	Y	Y	Y	Y	9
Tamir et al.	Y	Y	Y	Y	Y	N	Y	Y	Y	8
Tola et al.	Y	U	Y	Y	Y	N	Y	Y	Y	8

Y = yes; N = no; U = unclear; JBI critical appraisal checklist for studies reporting prevalence data: Q1: was the sample frame appropriate to address the target population? Q2: were study participants sampled appropriately? Q3: was the sample size adequate? Q4: were the study subjects and the setting described in detail? Q5: was the data analysis conducted with sufficient coverage of the identified sample. Q6: were the valid methods used for the identification of the condition? Q7: was the condition measured in a standard, reliable way for all participants? Q8: was there appropriate statistical analysis? Q9: was the response rate adequate, and if not, was the low response rate managed appropriately.

**Table 2:** Study characteristics included in the systematic review of nurses' documentation practice in Ethiopia, 2023.

Author	Year	Region	Study area	Study design	Sample	Prevalence
M. G. Selamu and L. G. Selamnu [26]	2022	SNNPR	Hadiya	Cross-sectional	384	40.02
Kasaye et al. [24]	2022	Amhara	Amhara	Cross-sectional	407	47.20
Andualem et al. [25]	2019	Amhara	Gojjam	Cross-sectional	240	47.50
Kebede et al. [18]	2017	Amhara	Gonder	Cross-sectional	206	37.40
Gizaw et al. [14]	2018	Oromia	Jimma	Cross-sectional	391	48.60
Demsash et al. [23]	2022	Oromia	Illubabor	Cross-sectional	415	51.10
Tasew et al. [22]	2019	Tigray	Tigray	Cross-sectional	316	47.80
Tamir et al. [17]	2021	Somali	Harar	Cross-sectional	430	47.50
Tola et al. [21]	2017	Addis Ababa	Addis Ababa	Cross-sectional	111	84.00

**Table 3:** Factors associated with nurses' documentation practice of the systematic review and meta-analysis in Ethiopia.

Factors	OR	CI	*I* ^2^ (%)	*P* value	Significance level
Motivation	29.69	(12.8, 47.7)	90.9	*P* ≤ 0.001	Significance
Adequate nurse-to-patient ratio	4.18	(2.5, 20.59)	88.3	*P* ≤ 0.001	Significance
Availability of format	3.01	(2.03, 23.07)	93.9	*P* ≤ 0.001	Significance
Training	10.08	(2.44, 41.7)	80.9	*P* ≤ 0.001	Significance
Knowledge towards documentation	7.54	(4.06, 13.9)	0.00	0.906	Nonsignificance
Attitude	18.37	(2.78, 121.38)	60.3	0.081	Nonsignificance

## Data Availability

The datasets generated and/or analyzed during the current study are not publicly available to prevent any kind of misuse by the public before publication but are available from the corresponding author upon reasonable request.
